# Culture-Proven Disseminated and Meningeal Histoplasmosis Presenting as Septic Shock and Autoimmune Hemolytic Anemia in an Infant

**DOI:** 10.7759/cureus.8945

**Published:** 2020-07-01

**Authors:** Fabricio Sevilla-Acosta, Elisandro Jiménez-Cruz, Hazel Álvarez-Cabalceta, Rolando Ulloa-Gutierrez

**Affiliations:** 1 Pediatrics, Hospital Nacional De Niños "Dr. Carlos Sáenz Herrera", San José, CRI; 2 Pediatrics, Hospital La Anexión, Nicoya, CRI; 3 Microbiology Laboratory, Hospital La Anexión, Nicoya, CRI; 4 Pediatric Infectious Diseases, Hospital Nacional De Niños "Dr. Carlos Sáenz Herrera", San José, CRI

**Keywords:** disseminated histoplasmosis, septic shock, autoimmune hemolytic anemia, infants

## Abstract

Disseminated histoplasmosis is the most common clinical presentation of histoplasmosis in human immunodeficiency virus (HIV) negative infants from Costa Rica and Latin America. Initial presentation as septic shock and autoimmune hemolytic anemia is uncommon. Even more, detection of *Histoplasma capsulatum* by culture in peripheral blood and cerebrospinal fluid (CSF) is extremely rare. We describe the case of a three-month-old Costa Rican immunocompetent infant who presented with shock and hemolytic anemia secondary to disseminated histoplasmosis that was confirmed by bone marrow aspirate and positive peripheral blood and CSF cultures.

## Introduction

*Histoplasma capsulatum* is a dimorphic fungus endemic in certain areas of North, Central, and South America, Africa, and Asia. However, cases have been also reported in Europe. Outbreaks have been found in pigeon or chicken breeders in places where bats are common (caves) and in old demolition sites [1].

Disseminated histoplasmosis usually occurs in immunocompromised patients who reside in *H. capsulatum* endemic regions. However, in immunocompetent infants, it develops after exposure to a large or prolonged inoculum of the pathogen, resulting in case fatality rates as high as 40% to 50% [2]. It is most likely to occur in infants, the elderly, and immunosuppressed patients, especially those with acquired immunodeficiency syndrome (AIDS), cancer, or solid organ or bone marrow transplant. Of apparently immunocompetent individuals with disseminated histoplasmosis, 20% are children [2].

In disseminated disease, the highest culture yield is from bone marrow aspirates, being positive in more than 75% of cases [1]. Less commonly, organisms may be observed by fungal staining of sputum, sterile body fluids, or peripheral blood smears [2]. However, even in large studies, detection of *H. capsulatum* in peripheral blood is infrequent [3].

Central nervous system (CNS) involvement occurs in 5% to 10% of individuals with disseminated histoplasmosis [4,5]. There are few reports of CNS histoplasmosis in immunocompetent hosts, and only a small percentage of cases correspond to pediatric patients [6]. It is suggested that in children, CNS is involved in 62% of disseminated histoplasmosis cases [2,7,8]. The diagnosis can be established by antigen and antibody testing of the cerebrospinal fluid (CSF) and serum, and antigen testing of the urine in most patients [4]. Histoplasma species can be isolated from fungal culture of CSF in only one quarter of patients, and visualization of yeast is rare [4]. Even when CNS involvement is suspected, laboratory confirmation can be challenging. In two large reviews, CSF cultures were positive in only 28% of patients, and growth was delayed for several weeks after presentation [4,5]. Isolation of *H. capsulatum* from CSF or brain tissue is diagnostic; however, culture is insensitive, and slow growth may result in significant treatment delay [5]. While the sensitivity for culture is low, this test remains the gold standard for confirming the diagnosis of CNS histoplasmosis and may be the only basis for diagnosis in some cases [5].

Here we describe an uncommon and severe case of a Costa Rican infant with disseminated histoplasmosis confirmed by both peripheral blood and CSF cultures, an uncommon scenario in pediatric clinical practice.

## Case presentation

**Figure 1:**
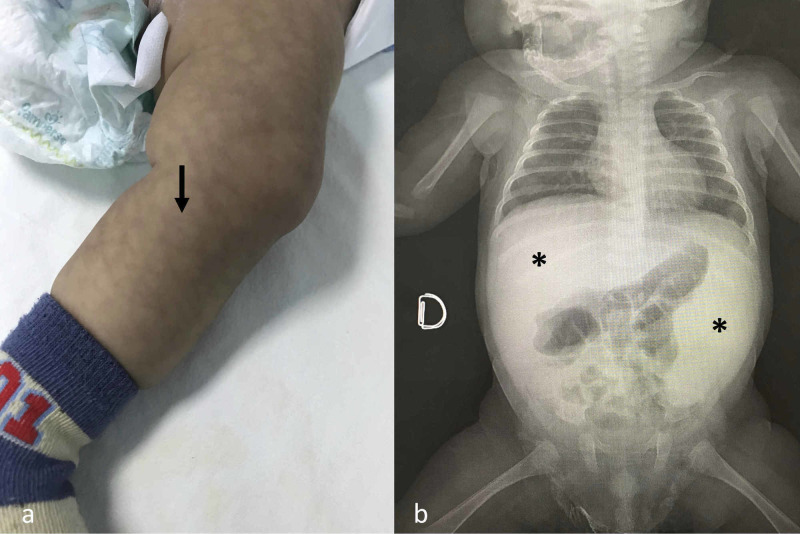
The patient on admission to the community hospital had (a) mottled skin with reticular aspect (arrow) and (b) severe hepatosplenomegaly also visible on abdominal plain X-ray film (asterisks).

A three-month-old Costa Rican male was admitted to a peripheral community hospital part of the social security system of Costa Rica (CCSS) because of two days of vomiting. On admission, physical examination revealed a febrile male with severe dehydration, mottled skin, and hepatosplenomegaly (Figure 1).

He needed initially 40 cc/kg of intravenous crystalloids. He persisted febrile with abdominal distention and developed a tonic seizure treated with one dose of intravenous diazepam. Samples were obtained for laboratory analysis, including peripheral blood cultures and CSF culture. Following a lumbar puncture, the patient had an apnea and decortication postures; therefore, he needed immediately endotracheal intubation, mechanical ventilation, and one dose of mannitol to reduce the intracranial pressure. A brain ultrasound revealed dense and lumpy material inside the lateral ventricles, an abdomen ultrasound confirmed the hepatosplenomegaly, and intra-abdominal free fluid was seen. Intravenous cefotaxime was started as an empiric therapy of septic shock.

A complete blood count was compatible with autoimmune hemolytic anemia (hemoglobin: 7.2 g/dL; reticulocytes: 9%; direct Coombs: ++ IgG and C3d) and thrombocytopenia (platelets: 60,000/mm3), with normal leukocytes count (5.300/mm3; absolute neutrophil count: 1,219). One dose each of intravenous immunoglobulin (1 g/kg) and methylprednisolone (30 mg/kg) was administered. Coagulation times and other hematological revealed disseminated intravascular coagulation (prothrombin time [PT]: 29%, activated partial thromboplastin time [ATTP]: 61 seconds, international normalized ratio [INR]: 2.18); therefore, reanimation was continued with fresh frozen plasma, red blood cells, and cryoprecipitates. Of interest, acute inflammatory markers were negative: C-reactive protein (CRP) of 12 mg/L and procalcitonin of 0.5 mg/mL. Further laboratory investigations revealed normal blood urea nitrogen, creatinine, electrolytes, and liver enzymes. CSF analysis revealed normal glucose (79 mg/dL), elevated proteins (845 mg/dL), lymphocytic pleocytosis (leukocytes: 600/mm3; 85% lymphocytes; 15% neutrophils), and cerebral bleeding (3 000 erythrocytes), and no bacteria or fungal forms seen on gram stain. He developed refractory septic shock; therefore norepinephrine and epinephrine were started and the patient was transferred to the only national tertiary pediatric center of the country.

On admission, the child continued on refractory septic shock, had pancytopenia, and coagulopathy, and was transferred to the pediatric intensive care unit. Under the suspicion of macrophage activation syndrome, a bone marrow aspirate was performed; blastospores of *H. capsulatum* were seen at direct microscopy, but no hemophagocytosis was observed. Polymerase chain reaction (PCR) for *H. capsulatum* in bone marrow aspirate was negative, and culture of bone marrow was not requested. Samples from blood and CSF that initially were cultivated in conventional aerobic bacterial cultures were centrifugated and planted onto Sabouraud’s dextrose agar and Mycosel agar at 35-37°C.

**Figure 2:**
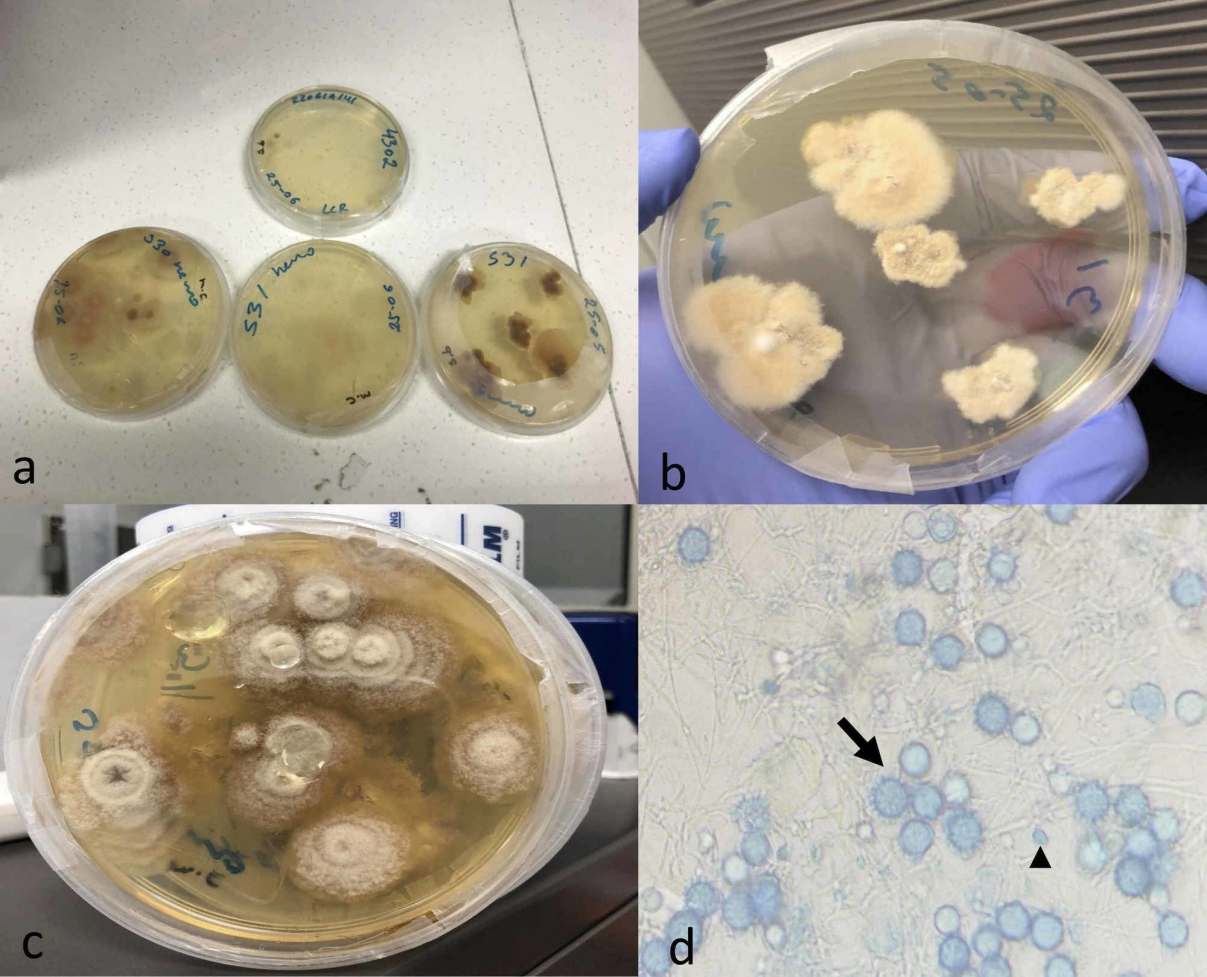
Cultures of peripheral blood and CSF. (a) Fungal cultures of peripheral blood and CSF in Sabouraud’s dextrose agar and Mycosel agar. (b,c) Macroscopic growing of *Histoplasma capsulatum* filamentous colonies. (d) Tuberculate macroconidia of 8-15 µm (arrow) and microconidia of 2-4 µm of diameter (arrowhead) characteristic of *H. capsulatum* colonies in blue lactophenol. CSF, cerebrospinal fluid

Intravenous deoxycholate amphotericin B was initiated immediately (1 mg/kg daily) and extended for one month. At the end of four weeks, both the peripheral blood and CSF cultures obtained at the referral community hospital were positive for *H. capsulatum* (Figure 2).

The patient recovered completely with no sequelae. Immunologic studies of the child including HIV, immunoglobulins (IgA, IgG, IgM), nitro blue tetrazolium test (NBT test), and flow cytometry were all normal. A large inoculum of bat guano was located at his home in a rural place of Costa Rica.

## Discussion

In pediatric patients, the clinical forms of histoplasmosis vary according to age group. In infants, the acute progressive disseminated form predominates (80%) [9]. Disseminated histoplasmosis usually presents clinically with fever, malaise, hepatosplenomegaly, and lymphadenopathy [10]. Severe disease can manifest as sepsis syndrome, hypotension, disseminated intravascular coagulation, acute renal failure, and respiratory distress [11]. CNS involvement occurs as a result of hematogenous dissemination to the meninges or the brain. The CSF changes in meningitis are similar to those noted for other fungal meningitides and tuberculous meningitis. Proteins are elevated, glucose is modestly low, and leukocytes count usually range between 50 and 500 cells/mm3, predominantly mononuclear cells [11]. In our case, the child presented with refractory septic shock, autoimmune hemolytic anemia, and coagulopathy. Rapid progressive histoplasmosis is not commonly reported in the literature, and factors that explain this progression are unknown to date, but the inoculum size and the immune response of the host could explain this rapid progression. The development of disease associated with the initial dissemination of *H. capsulatum* depends mainly on the host. Infants, presumably because of the immaturity of their cell-mediated immune system, are a special group that develop severe life-threatening disease when exposed to *H. capsulatum* [11].

Hemolytic anemia is not a common presentation of disseminated histoplasmosis. In our review of the literature, there are only three reported cases and none of these are children. In 2012, Chang et al. described a 65-year-old female with hemolytic anemia and a pulmonary nodule with pleural affection, pancytopenia, and elevated liver enzyme. Diagnosis was made by direct examination of sputum and bone marrow culture [12]. A second case was described by Landaeta et al. in a 70-year-old female [13]. Chejara et al. reported a third case in 2016 in a 30-year-old male diagnosed by liver and bone marrow biopsy [14]. It is possible that among some pediatric disseminated histoplasmosis case series, there have been infants with hemolytic anemia. However, to the best of our knowledge, our patient represents the first case report in the literature.

Isolation of *H. capsulatum* in cultures taken from peripheral blood and CSF is uncommon. One of the reasons is that these patients commonly present with severe thrombocytopenia and coagulopathy, and therefore lumbar puncture cannot be performed or is deferred. Samples of tissue or body fluids that are sent to the laboratory for culture are plated onto Sabouraud’s dextrose agar and incubated at 25°C to allow for growth of the mycelial phase of *H. capsulatum*. The lysis-centrifugation system has been shown to be more sensitive than automated systems for growing *H. capsulatum* from blood. Cultures usually yield no growth from CSF, and the diagnosis almost always must be made in a different manner [11]. Wheat et al. evaluated the largest cohort of patients with CNS histoplasmosis in 2018. In this study, they determined that *Histoplasma* can be isolated from fungal culture of CSF in only one quarter of patients, and visualization of yeast is rare. Only in 38% of patients, *Histoplasma* is isolated from CSF culture, or yeast resembling *H. capsulatum* were observed (in brain tissue histopathology or in CSF) [4]. In this case, early performance of the lumbar puncture prior to severe coagulopathy and amphotericin B initiation contributed to culture confirmation despite a late growth.

In our case, the samples were taken initially from blood and CSF in conventional aerobic bacterial cultures and were negative. Four days later, when the suspicion of disseminated histoplasmosis was high enough because the blastopores of *H. capsulatum* were seen on bone marrow aspirate, we used the leukocytes concentration technique to isolate the *H. capsulatum*. CSF and blood were centrifugated at 2,500 rpm for 15 minutes. Centrifugation system was used to concentrate the samples and obtain the buffy coat. The buffy coat was then planted onto Sabouraud’s dextrose agar and Mycosel agar at 35-37°C to allow the growth of the *H. capsulatum*. Four weeks later, growth of the fungus was seen with this technique in both peripheral blood and CSF samples. In CNS histoplasmosis of immunocompetent hosts, inoculum size is the main determinant of clinical manifestations and isolation [6].

As detection of *H. capsulatum* by CSF culture is difficult, Bloch et al. [5] performed a retrospective multicenter study to evaluate the sensitivity and specificity of a new anti-*Histoplasma* antibody enzyme immunoassay (EIA) for the detection of IgG and IgM antibody in the CSF for the diagnosis of CNS histoplasmosis. In this study, they found that CSF *H. capsulatum* culture was positive in only 19% of cases with diagnosis of CNS histoplasmosis defined as a patient with CNS inflammation (defined as CSF, white blood cell count ≥ 5 cells/μL) or brain imaging abnormalities and supporting laboratory studies (culture of CSF or elsewhere, detection of *Histoplasma* antigen by EIA or anti-*Histoplasma *antibodies in the CSF by ID [immunodiffusion] or CF [complement fixation]). This study concludes that testing CSF for anti-*Histoplasma* IgG and IgM antibody complements antigen detection and improves the sensitivity for diagnosis of meningitis [5]. In our case, we did not use antigen or antibody detection in CSF, but we think that these methods could have even made the diagnosis of *Histoplasma* meningitis earlier.

Furthermore, even in large studies, detection of *H. capsulatum* in peripheral blood is infrequent [3]. Ouellette et al. conducted a single-center retrospective review of proven and probable cases of histoplasmosis in children aged 0 to 18 years between April 2008 and April 2014 [3]. In a six-year period study including 73 children with histoplasmosis, no routine or fungal blood cultures (91 total samples from 25 patients) grew *H. capsulatum* [3]. To our knowledge, their study is the largest contemporary retrospective evaluation of children diagnosed with proven or probable histoplasmosis. The highest yields of *H. capsulatum* from blood culture have been described in patients with disseminated histoplasmosis, chronic cavitary histoplasmosis, or acute pulmonary histoplasmosis after large inoculum exposure [3,11].

Odio et al. reported 40 patients with disseminated histoplasmosis in Costa Rica from 1983 through 1996 at our children’s hospital [2]. All patients were from endemic regions and presented with fever, spleen and/or liver enlargement, and hematologic abnormalities. Diagnosis was made by histology and culture of bone marrow, spleen, lymph node, bronchoalveolar, or liver samples. Fungal forms were seen in the smear of the spleen aspirate in 5/5 patients (100%), lymph node biopsy in 5/6 (83.3%), bone marrow aspirate in 30/40 (75%), bronchoalveolar secretions obtained by bronchoalveolar lavage in 9/12 (75%), and liver biopsy in 3/4 (75%). *Histoplasma* grew in cultures from at least one sample for each patient [2]. This is the largest case series of disseminated histoplasmosis in Costa Rica, and at that time no patient was diagnosed by blood and CSF cultures as compared with our case.

In other literature from Latin-American, López et al. reported 45 patients with histoplasmosis in Colombia, of which only two had *H. capsulatum* isolated from blood and eight from CSF [9]. In Argentina, Voto et al. reported 13 patients in a 10-year period, but none had *H. capsulatum* isolated in blood and only two had it isolated in CSF [8].

Progressive disseminated histoplasmosis in children is fatal if untreated. Amphotericin B deoxycholate (1 mg/kg daily) given for four weeks has been used successfully and with minimal toxicity. In contrast to cases in adults, meningitis that accompanies progressive disseminated histoplasmosis of infancy responds to amphotericin B deoxycholate (given for four to six weeks) without a high rate of relapse. Treatment recommended for progressive disseminated histoplasmosis of infancy does not need to be modified in patients found to have CNS involvement [15]. In our case, we used a course of four weeks of amphotericin B deoxycholate followed by a three-month oral course of itraconazole. Currently, the patient is asymptomatic and has no evidence of relapse or sequelae.

## Conclusions

Herein, we described the case of a three-month-old boy with disseminated histoplasmosis who on admission presented in septic shock and developed autoimmune hemolytic anemia, a rare manifestation in children with disseminated histoplasmosis. Although disseminated histoplasmosis is a rare cause of septic shock, physicians should be aware of this possibility in endemic areas in the differential diagnosis of infants with fever, malaise, hepatosplenomegaly, and abnormalities in the complete blood count, especially pancytopenia or any other cytopenia. Culture-proven histoplasmosis from peripheral blood and CSF culture is extremely rare, but if there is high suspicious of histoplasmosis, blood and CSF fungal cultures should be obtained. Using the centrifugation technique in the laboratory increases the possibility of isolation and confirmation.
